# Hypercalcemia as a rare presentation of hyaline fibromatosis syndrome from different Sudanese families: two case reports

**DOI:** 10.1186/s13256-023-03927-9

**Published:** 2023-06-02

**Authors:** Mariam M. Ismail, Salwa A. Musa, Samar S. Hassan, Mohamed A. Abdullah

**Affiliations:** 1 Pediatric Endocrinology Department, Gaafar Ibn Auf Children’s Hospital, Khartoum, Sudan; 2 Department of Paediatrics and Child Health, Faculty of Medicine, Al-Neelain University, Khartoum, Sudan; 3 Department of Paediatrics and Child Health, Faculty of Medicine, University of Khartoum, Khartoum, Sudan

**Keywords:** Hyaline fibromatosis syndrome, Hypercalcemia, Case report

## Abstract

**Background:**

Hyaline fibromatosis syndrome is a rare progressive autosomal recessive connective tissue disorder caused by a mutation in the *ANTXR2/CMG2* gene. According to its severity, patients may present with skin nodules or visceral infiltration, which carries a poor prognosis. Hypercalcemia has not been reported as a presenting feature of this syndrome. Stimulation of osteoclasts by inflammatory factors and immobilization­-induced hypercalcemia have played role in the pathophysiology. To our knowledge, this is the first report of hypercalcemia-associated hyaline fibromatosis syndrome.

**Case presentation:**

Here, we describe cases of two Sudanese patients, a boy aged 9 months and a girl aged 3.5 years with hypercalcemia as an associated presenting feature of hyaline fibromatosis syndrome. Other features include gingival hypertrophy, painful joint swellings, and restriction of movement, which was misdiagnosed as juvenile rheumatoid arthritis. Workup showed normal phosphate, normal to mildly elevated parathyroid hormone, low vitamin D 25. Genetic testing confirmed the mutation of the *ANTXR2/CMG2* gene. Both patients responded well to medical therapy for hypercalcemia, but one of them with the severe form of juvenile hyaline fibromatosis died due to sepsis, while the other one has maintained normocalcemic status.

**Conclusions:**

These cases highlight the rare presentation of this syndrome and reflect the importance of biopsy and genetic testing in reaching the diagnosis, especially when the clinical presentation can mimic other inflammatory bone disorders. Calcium levels should be checked in such cases.

## Introduction

Hyaline fibromatosis syndrome or (juvenile hyaline fibromatosis) is a rare progressive autosomal recessive disorder that involves the connective tissue. It was first described by Murray in 1873 and termed molluscum fibrosum [1]. It is caused by genomic sequence variations in the *ANTXR2/CMG2* gene [2]. Two forms of the disease exist; juvenile hyaline fibromatosis (JHF) and hyaline fibromatosis syndrome (HFS), which is another term for infantile systemic hyalinosis (ISH) [3]. JHF and ISH are allelic, and JHF has a more benign course while ISH is a more serious form with an early onset and systemic involvement that can exist since birth. It commonly infiltrates the small intestine and colon, leading to malabsorption and protein-losing enteropathy, with diarrhea, failure to thrive, growth failure, increased susceptibility to infections, and normal cognitive abilities [4, 5]. The disease severity ranges from mild to severe, Nofal *et al*. recommended a grading system of HFS and this was adjusted by Denadai *et al*., who categorized it into grade 1 or mild (skin and/or gingival involvement), grade 2 or moderate (joint and/or bone involvement), grade 3 or severe (internal organ involvement with or without clinical manifestations), and grade 4 or lethal (severe clinical decompensation) [6].

In this report, we present two cases of HFS from two different families, with unusual presentation of hypercalcemia in addition to skin and skeletal manifestations of the disease. Genetic tests confirmed *ANTXR2* gene mutation. No similar cases of HFS have been reported before from sub-Saharan Africa, and this is the first report of hypercalcemia-associated HFS.

### Case 1

A 9-month-old Sudanese boy was referred to our department as a case of hypercalcemia. He was born at term following normal pregnancy and labor with low birth weight. He was the first baby to a healthy first-degree related healthy parents. Irritability while handling, painful pigmented swellings over knuckles, and progressive joint deformities (elbows, wrists, hips, knees, and ankles) were noticed since the second week of life. That was followed by recurrent infections, oral thrush, and diarrhea in the following 6 months. In addition, he had constipation, vomiting, poor feeding, failure to gain weight, and distinct impaired gross motor and fine motor development. There was no polyuria, seizures, or fractures. He was diagnosed as a case of rheumatoid arthritis and treated with corticosteroids for 6 months without improvement. On routine workup he was found to have hypercalcemia with a calcium level of 18 mg/dl [4.49 mmol/l, alkaline phosphatase 93 U/l, and parathyroid hormone (PTH) 69.1 pg/ml (10–65 pg/ml)] but serum phosphate was not checked. He was then referred to our unit for further evaluation.

**Fig. 1:**
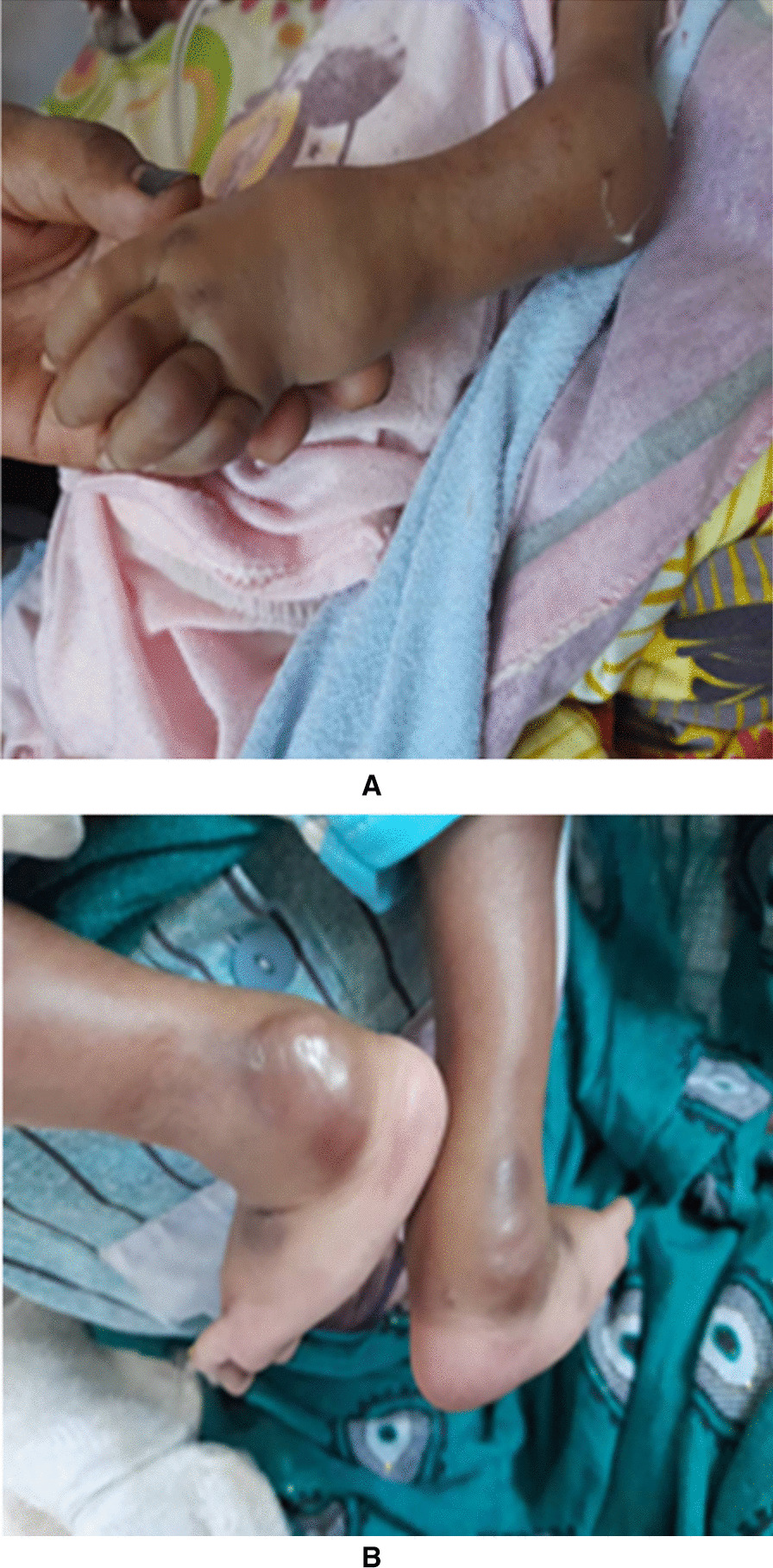
**A** Swellings over the left elbow, wrist, and fingers. **B** Ankles swellings

**Fig. 2:**
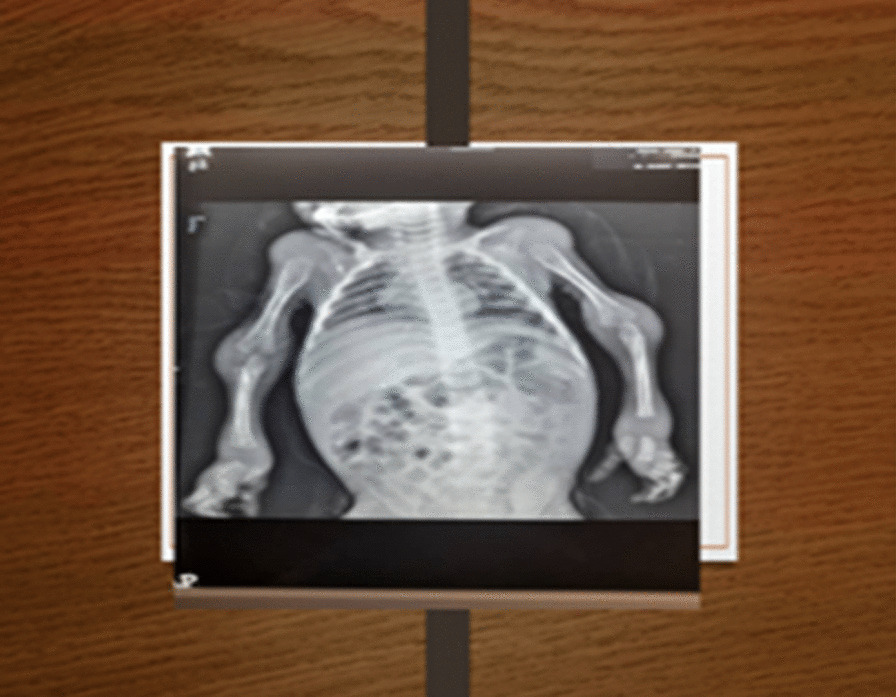
X-ray shows osteopenia and subcutaneous calcifications over shoulders, elbows, wrists, and fingers

Examination at presentation revealed nondysmorphic hypotonic child with gingival hypertrophy, umbilical hernia, and no organomegaly. There were swollen, deformed joints with hyperpigmented nodules at elbows, wrists, fingers, knees, and ankles (Fig. 1). They were tender with limited joint movements and diffuse, tender erythematous swelling at the lumbosacral region. His weight was 4.7 kg (−4 SD), length 60 cm (−3 SD), and head circumference 42.2 cm (third centile). He had a wide anterior fontanel (7 × 6 cm). His weight for length was < −3 SD and his middle upper arm circumference (MUAC) was 9 cm, indicating severe acute malnutrition. Laboratory studies showed serum calcium of 18 mg/dl (4.49 mmol/l), phosphate of 2.5 mg/dl (0.8 mmol/l), PTH 166 pg/ml (15–65 pg/ml), and a vitamin D level of 8.61 ng/ml (< 10 denotes deficiency). There were no facilities to measure PTHr levels in our setting. His urinary calcium creatinine ratio was 0.062 (up to 0.6). His erythrocyte sedimentation rate (ESR) was 10 mm /hour and C-reactive protein (CRP) was 1.9 mg/dl. Urine culture revealed *Escherichia coli*. Hemoglobin was 8.7 g/dl, total leukocyte count was 14,800/cumm (neutrophils 56.3%), a platelet count of 960,000/cumm, and normal renal function tests with hyponatremia and hypokalemia. Radiographs of long bones showed osteopenia (Fig. 2). Abdominopelvic ultrasound demonstrated features of chronic renal parenchymal disease, medullary nephrocalcinosis, and multiple nonobstructing renal stones. Neck ultrasound showed small lower cervical lesions suggesting either thyroid nodule, parathyroid adenoma, or lymph node. Nuclear scanning was considered but was not available. It was not possible to do histopathology due to financial reasons but genetic testing at Centogene laboratories in Germany revealed a homozygous mutation in the *ANTXR2* gene. He was managed with intravenous fluid and rehydration solution for malnutrition (ReSoMal), furosemide, bisphosphonate, and antibiotics. Then his serum calcium dropped to 10.3 mg/dl (2.57 mmol/l) and he maintained normocalcemia. Unfortunately, he died soon after readmission due to sepsis before rechecking of the PTH.

## Case 2

A 3.5-year-old Sudanese girl was referred to our clinic for evaluation of poor growth and subcutaneous nodules. She was born to healthy first-degree consanguineous parents at term with a low birth weight after an uncomplicated pregnancy. At 6 weeks, her mother noticed irritability on handling the child. She was then admitted with unexplained asymptomatic hypercalcemia at the age of 6 months. By 9 months, the child developed painful tissue swellings on the scapulae and small joints of the hands, with restriction of movements that progressed over time to involve her jaws, knees, elbows, ankles, skull, neck, buttocks, and perianal area. This was in addition to poor feeding, failure to thrive, and delayed gross motor milestones. Her calcium level was found to be 14.7 mg/dl (3.6 mmol/l), phosphate 4.2 mg/dl (1.3 mmol/l), alkaline phosphatase 152 μ/l, and PTH 20 pg/ml (3.6–32 pg/ml), which was treated by intravenous fluid and furosemide. She was not on vitamin D. She was then referred to a rheumatologist for the joint swellings. She was diagnosed as having juvenile idiopathic arthritis and received corticosteroids and methotrexate for 29 months without improvement, and thus referred to us to exclude a metabolic disorder. The patient had three other healthy siblings. Her calcium level was rechecked after admission in our unit and was 10 mg/dl (2.4 mmol/l).

**Fig. 3:**
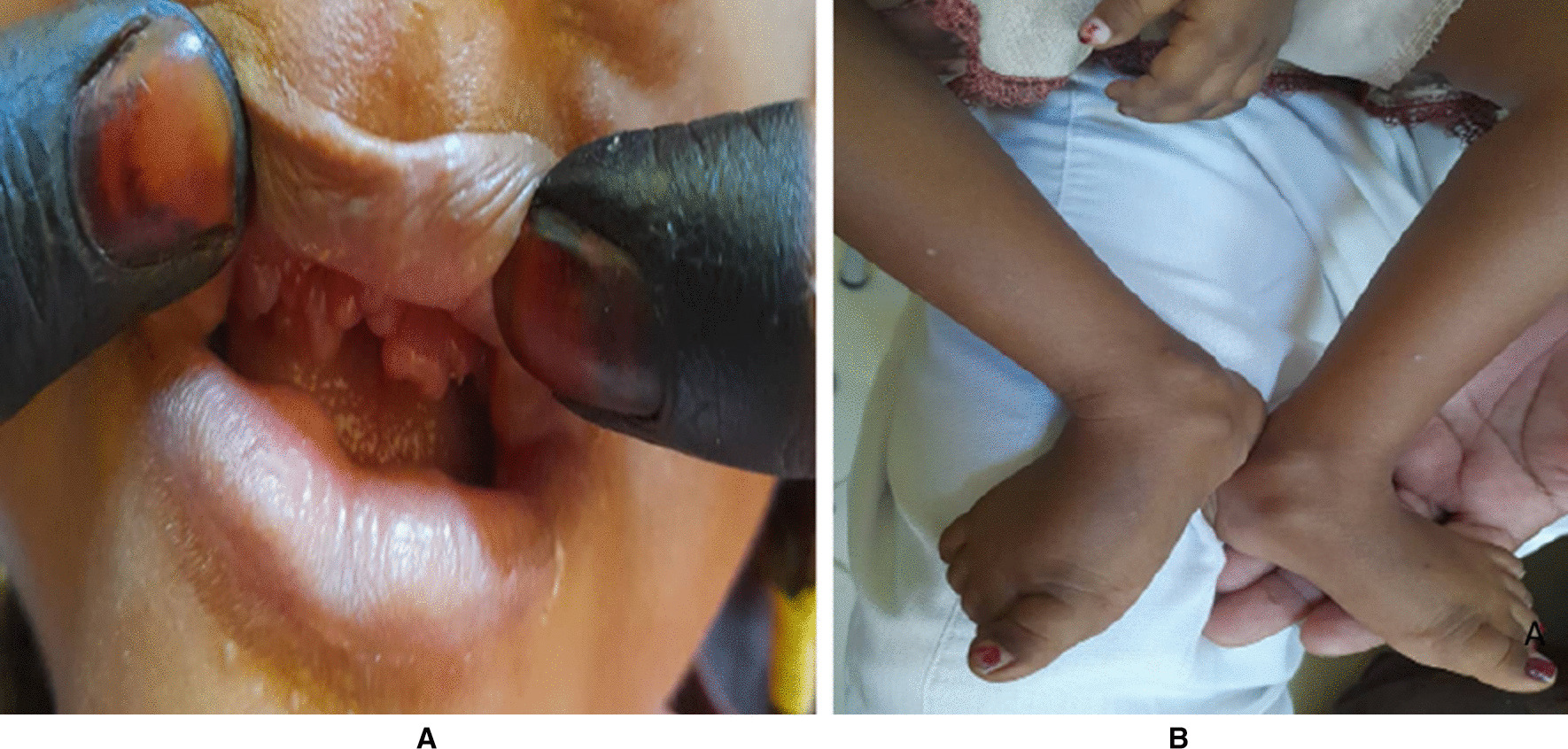
**A** Gingival hypertrophy, **B** Swellings over fingers, toes, and ankles

Examination at 6 months old showed normal physical examination with no dysmorphic features: weight 6.9 kg (25th percentile), length 61 cm (5th percentile), and head circumference 41.8 cm (10th percentile). At the age of 3 years she had gingival hypertrophy (Fig. 3A) and papules in the tip and around the nose, skull, neck, buttocks and perianal area, lower jaw, anterior and posterior part of the neck, interphalangeal joints, elbows, and ankles (Fig. 3B) that led to deformities associated with tenderness and decreased range of movement. There were no overlying skin changes.

**Fig. 4:**
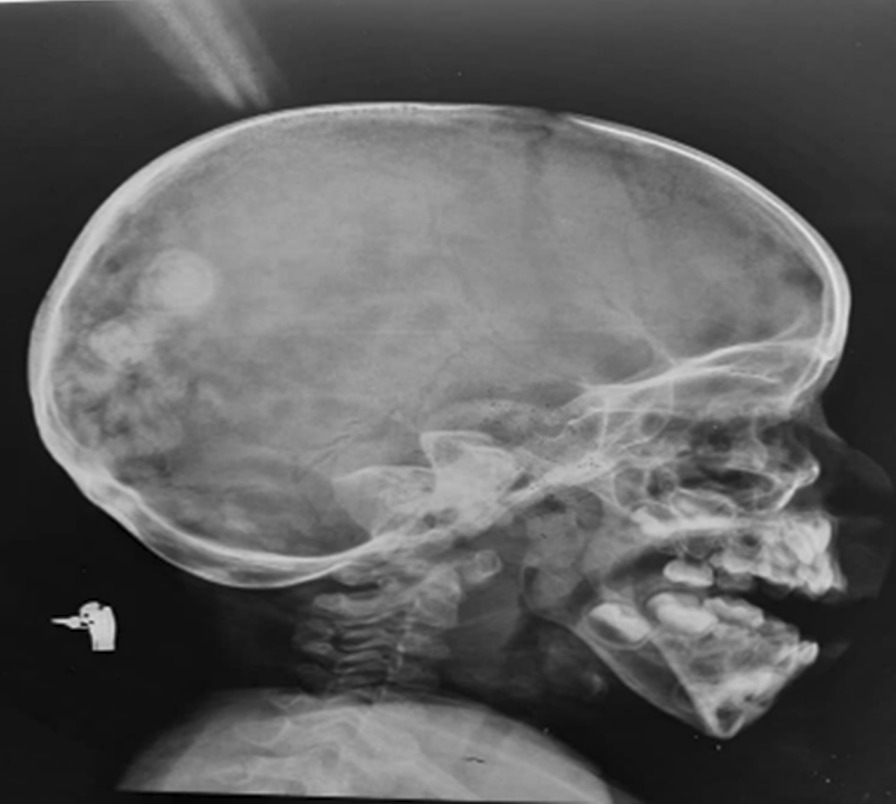
X-ray shows subcutaneous calcifications over the skull

Laboratory tests revealed serum calcium of 14.5 mg/dl (8.5–10.8 mg/dl), urinary calcium: creatinine 0.39 (˂ 0.2), serum phosphorus of 4.7 mg/dl equivalents to 1.5 m mol/L, PTH was 19.79 pg/ml (3.6–32 pg/ml), 25 hydroxy vitamin D level was 15 ng/ml (30–100 ng/ml), 1,25 dihydroxy vitamin D 78 pg/ml (20–54 pg/ml), urea 13 mg/dl (15–48.5 mg/dl), creatinine 0.1 mg/dl (0.31–0.47 mg/dl), sodium 140 mmol/l (136–145 mmol/l), and potassium 4.6 mmol/l (3.5–5.1 mmol/l) with normal liver function. X-rays of the limbs and skull showed subcutaneous calcifications (Fig. 4) and osteopenia and she was thus initially thought to have tumor calcinosis. No biopsy was done. Genetic testing done at Centogene laboratories confirmed a a homozygous mutation in the *ANTXR2* gene. She was managed through a multidisciplinary approach. She maintained normocalcemia and normal PTH, as well as ALP lover the last 3 years. Her last serum calcium was 10 mg/dl (2.4 mmol/l), with weight 10 kg (−5 SD), length 81 cm (−5 SD), and head circumference of 47.5 cm (25th percentile). She was referred for physiotherapy and dental care. She had a restriction of joint movement including jaws that led to poor feeding. The family had been counseled regarding her condition. Our patients’ characteristics are presented in Table 1.

**Table 1:** Characteristics of the cases

Characteristic		Case 1	Case 2
Age (months)		9	6
Gender		Male	Female
Presentation		Poor feeding, failure to gain weight, vomiting, constipation, bone deformities, restriction of joints movement, and hypercalcemia	Irritability on handling and hypercalcemia
Progression T0	Ca (mg/dl)	18	14.7
	PO_4_ (mg/dl)		4.2
	ALP(U/l)	93	152
	PTH (pg/ml)	69.1 (10–65)	20 (3.6–32)
	Urinary calcium		0.39
T1	Ca (mg/dl)	10.3	9.9
	PO_4_ (mg/dl)	2.5	3.8
	Vitamin D	8.61	
	PTH (pg/ml)	166 (15–65)	
	Urinary calcium	0.062 (up to 0.6)	
T2	Ca (mg/dl)	8	
T3	Ca (mg/dl)		10
	PO_4_ (mg/dl)		3.8
	PTH (pg/ml)		19.79 (3.6–32)
	25 OH Vitamin D (ng/ml)		15 (30–100)
	1,25 OH Vitamin D (ng/ml)		78 pg/ml (20–54)
Outcome		Death	Maintained normal calcium

T0: initial presentation; T1: follow-up after 48 hours; T2: follow-up after 1 week; T3: follow-up after 3 years; Ca: Calcium; Po4: Phosphorus; ALP: Alkaline phosphotase; PTH: parathyroid hormone

## Discussion

Hyaline fibromatosis syndrome (HFS) is an autosomal recessive disorder due to deposition of hyalinizing fibrosis. Less than 120 cases of JHF have been published worldwide [7]. JHF has no sex predilection and is common in Turkish, Indian, and Moroccan populations, where interfamilial marriage is common [1]. In our cases, both were children of consanguineous parents, which is commonly seen in this pattern of inheritance. This is the first case of HFS reported from sub-Saharan Africa, and the second report of HFS from Sudan [2] but the first with an associated hypercalcemia.

Pathophysiology of HFS is not well known, but it is believed that it could be due to increased synthesis of glycosaminoglycans by fibroblasts. Another explanation is an increase in type VI and III collagen, besides increased degradation of type I collagen [2]. It is well known that stimulation of osteoclast proliferation and maturation by tumor necrosis factor-α, interleukin-6, and other inflammatory factors results in increased blood calcium [8]. In these cases, hypercalcemia is PTH independent. Case 1 presented with hypercalcemia and medullary nephro-calcinosis with multiple nonobstructing renal stones with mildly elevated PTH, and the patient died before rechecking it. These features had never been described before in the literature. Furthermore, case 2 had a different presentation with bone pain and hypercalcemia before the appearance of subcutaneous nodules, with normal PTH over the past 3 years. Hypercalcemia seems to be a secondary rather than primary finding and it mimics subcutaneous fat necrosis [9]. Both patients had prolonged immobility due to pain and restriction of joints movement. Immobilization causes an imbalance of osteoblast-mediated bone formation and osteoclast-mediated bone absorption, which eventually leads to hypercalcemia [10]. Further studies are needed to find out the causes of hypercalcemia in HFS.

HFS presents with various symptoms, including thickened skin with nodules, papules, and plaques, often with a periorificial and perianal distribution, gingival hypertrophy, osteopenia, and joint contracture. This is in addition to feeding problems, protein-losing enteropathy, malnutrition, and a predisposition to respiratory infections and diarrhea [11, 12]. Furthermore, it has been described that HFS can affect other organs such as the heart, trachea, and thyroid and adrenal glands [4]. In case 1 cortisol was normal and as the patient was sick, his thyroid function test was not checked, while case 2 had normal thyroid function test but we did not check cortisol levels.

Due to the rarity of this syndrome and its similar presentation to juvenile idiopathic arthritis, these cases were missed at presentation and treated as cases of rheumatoid arthritis with corticosteroids and methotrexate for a long time without response. The second case was thought to have a tumor calcinosis in view of the nodules and calcifications, and previously similar cases have been treated as such [13]. However, genetic testing confirmed HFS and a biopsy of the lesion was not performed. Farber’s disease is one of the differential diagnosis of HFS, as it shares some clinical features with HFS such as subcutaneous nodules and joint contractures; however, in Farber’s disease, hoarseness of voice is one of the main features [14, 15]. Diagnosis of HFS is supported by microscopic examination of nodule biopsy specimens, which reveals spindle-shaped fibrous cells with an abundance of broadly hyalinized matrices; however, neither of our cases underwent biopsy of the nodules, as genetic testing revealed the diagnosis, confirmed by the presence of the *ANTXR2* gene [1].

There is currently no successful treatment for HFS; supportive care is regarded as the mainstay choice of treatment [6]. Nonsteroidal antiinflammatory drugs, gabapentin, and opiates are given for pain relief, as well as bisphosphonates for osteoporosis and osteolysis [4]. Gingivectomy and dental care are essential to ensure strict oral hygiene, and surgical resection of the tumors and nodules may be required [13]. Physiotherapy has a crucial role in treating the joint stiffness, and local (triamcinolone injection) and systemic corticosteroids can be used with limited success, as well as penicillamine and methotrexate [2, 16, 17]. The leading cause of death is recurrent respiratory infections as well as intractable diarrhea, recurrent infections, and organ failure [18]. The median age of death of grade 4 cases is 15.0 months, and our first patient who had grade 4 died at the age of 9 months. This is contrary to what happened in our second case who had grade 2 disease and is alive but with a poor quality of life.

## Conclusions

This is the first report of hypercalcemia associated with HFS. The mechanism is not fully understood, thus further studies are needed to explain the link. Genetic testing can confirm the diagnosis and guide counseling for affected families to prevent disease in future generations. Treatment is largely supportive and a multidisciplinary team approach is crucial for a better outcome.

## Data Availability

Not applicable.

## Abbreviations

HFS: Hyaline fibromatosis syndrome
PTH: Parathyroid hormone
